# Effect of flap design and duration of surgery on acute postoperative symptoms and signs after extraction of lower third molars: A randomized prospective study

**DOI:** 10.15171/joddd.2017.028

**Published:** 2017-09-20

**Authors:** Nicola Mobilio, Renata Vecchiatini, Michele Vasquez, Giorgio Calura, Santo Catapano

**Affiliations:** ^1^Dental School, Dental Clinic, University of Ferrara, Italy

**Keywords:** Oral surgery, postoperative pain, third molar

## Abstract

***Background.*** Different surgical variables are assumed to play a role in postoperative course after lower third molar extraction. The aim of study was to assess whether flap design and duration of surgery can influence acute postoperative symptoms and signs after lower third molar extraction.

***
Methods.*** Twenty-five patients scheduled for lower third molar extraction were included in this study and randomly assigned to two groups in terms of flap design: group A (envelope flap) and group B (triangular flap). Swelling and trismus were assessed before and after surgery on days 0, 2 and 7. Pain was assessed for seven days after surgery. Maximum postoperative pain was chosen as the main outcome variable. ANOVA was used to assess differences between the groups regarding maximum postoperative pain, trismus and swelling at 2- and 7-day intervals. Pearson's correlation coefficient was used to assess correlation between duration of surgery and postoperative symptoms and signs.

***
Results.*** No significant difference was found between the two flap designs for any postoperative symptoms and signs. The duration of surgery was found to be correlated with both trismus (r = -0.44, P = 0.04) and swelling (r = 0.59, P = 0.004) as assessed 2 days after surgery. No associations were found between duration of surgery and maximum postoperative pain and trismus and swelling at 7-day interval.

***
Conclusion.*** Within the limits of the present study, the duration of surgery, and not the flap design, affected the acute postoperative symptoms and signs after lower third molar extraction.

## Introduction

The surgical extraction of lower third molar is a routine event in oral surgery and it is frequently associated with considerable postoperative adverse effects. Among acute complications are symptoms, like pain, and signs, like swelling and trismus.^1,2^ It is known that many surgical variables, like flap design or duration of intervention, can affect postoperative experience after lower third molar extraction, but different studies have presented different results. Indeed, it is widely recognized that increasing the operation duration results in more postoperative morbidity.^1,3,4^ Otherwise, the impact of flap design on acute postoperative symptoms and signs is less clear. Many studies found a different postoperative course in terms flap design, with the less extended flap generally being the one with fewer complaints.^5-13^ However, some studies failed to find any differences in postoperative symptoms and signs using different flaps.^14-18^

The aim of this study was to assess whether flap design and duration of surgery can influence acute postoperative symptoms and signs after lower third molar extraction.

## Methods

### Patients

Twenty-five medication-free otherwise healthy consecutive patients (18 women and 7 men; mean age: 27.88±9.75 years, age range: 18‒61 years) scheduled for lower third molar extraction on an ambulatory basis were included in this study.

All the patients presented complete mucosal inclusion of the third molar, and no previous or current inflammation or pain was reported in that area. Exclusion criteria consisted of age <18 years, diagnosed psychiatric disorders, diagnosed neurological diseases, diagnosed impaired communicative or cognitive abilities, contraindications to nonsteroidal antiinflammatory drugs (NSAIDs) or amoxicilline.

The study was designed according to the Declaration of Helsinki and approved by the Local Ethics Committee. Each patient provided a written informed consent for participation.

### Surgery and pharmacology

**Figure 1:**
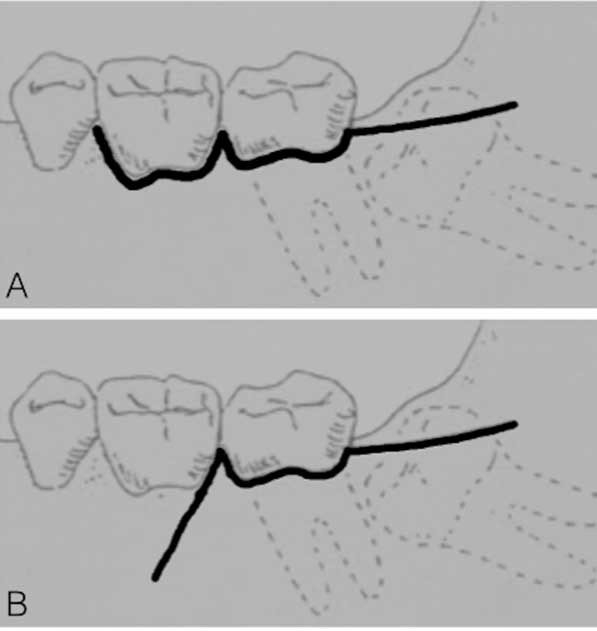
The two flap designs: envelope (A) and triangular (B).

All the surgical interventions were performed by the same dentist according to standard surgical and anesthetic protocols used at the dental clinic. Mepivacaine (2%) containing 1:100,000 adrenaline was administered as the inferior alveolar, buccal and lingual nerve block. The patients were randomly assigned to two groups in terms of flap design: group A (envelope flap) and group B (triangular flap). The division was made in order to obtain two homogeneous groups for gender and age. In group A, a sulcular incision was performed buccally from the first to the second mandibular molar with a distal incision along the mandibular ramus. In group B, an incision was performed from the mandibular ramus to the distobuccal aspect of the second molar. Then it became a sulcular incision up to the distobuccal edge of the first molar, where a releasing incision was made (Figure 1). Apart from the incision, the intervention was the same for the two groups. Lingual tissues were retracted and protected, the buccal and distal bone was removed with burs, tooth sectioning was performed with burs where necessary, and sutures were placed to achieve a primary or secondary closure, as appropriate. No medications were taken before tooth extraction. “Duration of surgery” was defined as the time from flap elevation until the end of suture.

Standard postoperative instructions were provided and standard analgesics were prescribed (ketoprofen 80 mg: 1st dose after 2 hours, 2nd after 8 hours, then 3 times a day for day 2 and 3; 0.12% chlorhexidine mouthrinse was prescribed from day 2 until day 7). A postoperative meeting was scheduled on days 2 and 7 to check swelling and trismus. During the second appointment the sutures were removed.

### Postoperative assessment

**Figure 2:**
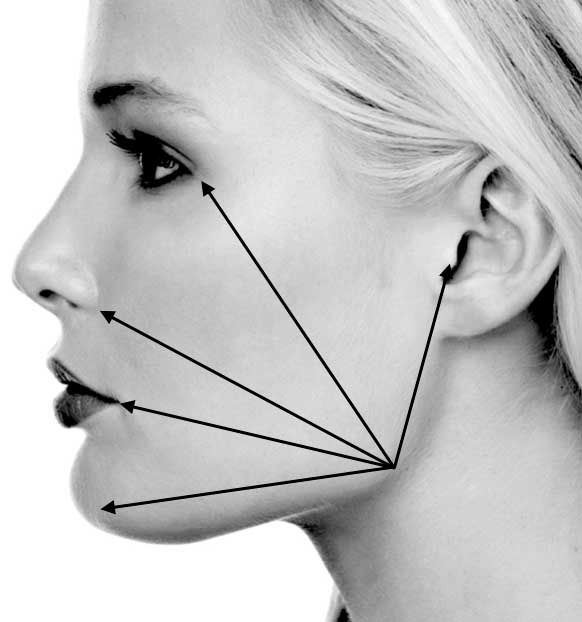
Schematic representation of points for measuring swelling.

Swelling and trismus were assessed by the third examiner before and after surgery, on days 0, 2 and 7. To assess swelling, 5 distances (in mm) through 6 facial points (angle of the mandible to tragus, to eye outer canthus, to labial commissure, to nasal border and to soft pogonion) were measured, and then the average percentage value was obtained as previously described (Figure 2).^3^

To assess trismus (represented by maximum intercisal opening [MIO] reduction) the distance between the incisal edges of the upper and lower central incisors was measured in mm 3 times each day. The differences between initial MIO and 2-day MIO and initial MIO and 7-day MIO were assumed as trismus on days 2 and 7, respectively.

Pain assessment was based on self-reported registrations on a 100-mm VAS, starting at the end of surgery and during the following 7 days at different hours. On the day of the surgery: every hour until the 10th postoperative hour; during the 1st and 2nd day after surgery at 8, 12, 16, 20 h; during the 3rd, 4th, 5th, 6th days after surgery at 20 h.

The patients were instructed to record daily pain assessments and NSAID requirements in a specific diary. Maximum postoperative pain was chosen as the main outcome variable, as previously reported.^19^

### Statistical analysis

Data were statistically analyzed using SPSS 22 for Mac OSX (IBM, Armonk, NY, USA). Kolmogorov-Smirnov test was used to confirm the normality of data distribution. Levene’s test was used to test the homogeneity of variances. Differences between the flap designs regarding maximum postoperative pain and trismus on days 2 and 7 and swelling on days 2 and 7 were analyzed with one-way ANOVA. Pearson's correlation coefficient was used to assess the correlation between the duration of surgery and postoperative symptoms and signs. P<0.05 was considered statistically significant.

## Results

Table 1 presents means (and standard deviations) of postoperative symptoms and signs in the two groups. No significant difference was found between the two flap designs for any postoperative symptoms and signs.

**Table 1:** Means (and standard deviations) of postoperative signs and symptoms in the two groups

**Variables**	**Groups**	
	**Group 1**	**Group 2**
**Subjects (number)**	13	12
**2-days trismus (mm)**	-22.33 (12.18)	-16.29 (4.23)
**7-days trismus (mm)**	-9.41 (8.23)	-7.13 (8.93)
**2-days swelling (%)**	6.6 (5.57)	9.97 (5.98)
**7-days swelling (%)**	2.12 (1.93)	1.17 (3.06)
**Maximum post-operative pain (100-mm VAS)**	57.06 (22.08)	48.75 (30.32)

**Figure 3:**
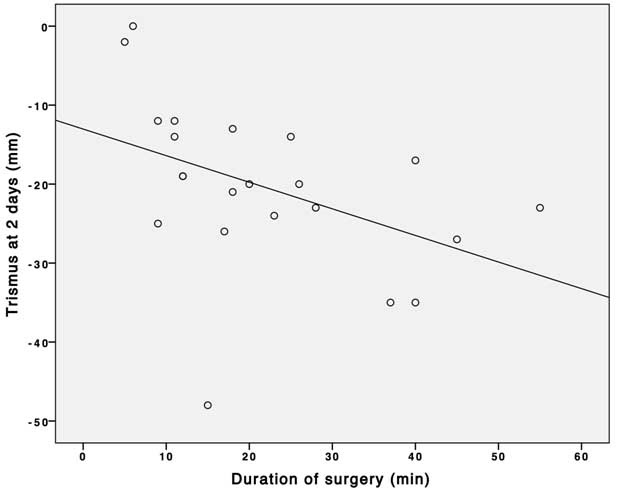
Correlation between the duration of surgery and trismus after 2 days.

**Figure 4:**
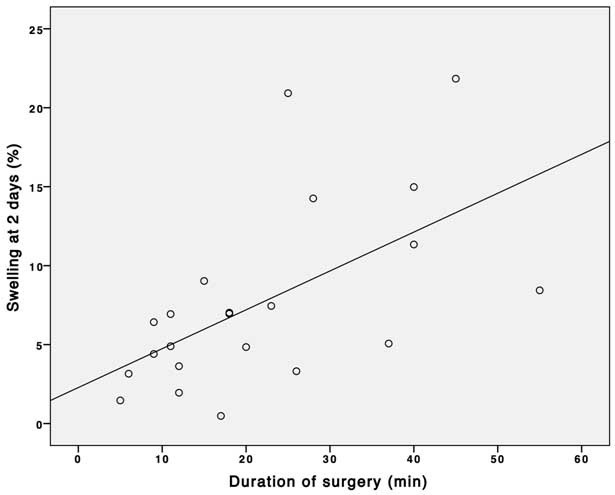
Correlation between the duration of surgery and swelling after 2 days.

The duration of surgery was found to be correlated to both trismus (r = -0.44, P = 0.04, Figure 3) and swelling (r = 0.59, P = 0.004, Figure 4) as assessed 2 days after third molar removal. No associations were found between the duration of surgery and maximum postoperative pain and trismus and swelling during the 7-day postoperative interval.

## Discussion

Lower third molar extraction is frequently associated with postoperative morbidity. Different surgical techniques have been introduced to perform less invasive intervention and, therefore, reduce postoperative symptoms and signs. In this view, less extended flaps have been proposed. In the present study, two different flap designs were compared: a simple intrasulcular envelope flap without a releasing incision and a triangular flap with a releasing incision. The triangular flap generally achieves a better and wider view during surgery, but because of the releasing incision, it is assumed to produce more inflammatory edema and therefore more postoperative signs. Indeed, many studies have reported a worst postoperative course when extended flaps are applied.^8-13^ In the present study, however, no differences were found in symptoms and signs in terms of flap design, consistent with other studies reported in the literature.^15-18^

The most important variable for postoperative discomfort appeared to be the duration of surgery. Such a result is widely reported in the literature. A study found an association between duration of surgery and postoperative symptoms and signs after third molar surgery.^4^ Such an association was not surprising: the longer the duration of tissue injury, the higher the amount of inflammatory mediators released; therefore, it could be a reflection of the severity of pain, swelling and trismus.

Some studies distinguish pain, which is subjective in nature, from trismus and swelling that can be objectively measured. Beyond the possibility of measuring it, pain is a more complex outcome to evaluate. Different from postoperative signs, pain is considered to be influenced by many factors, only partially explained by the surgical aspects. Many studies have found that inner charateristics like pain tolerance or pain expectation may play a role in subjective symptoms like pain.^19^ This may explain why in the present study no association was found between the duration of surgery and postoperative pain.

According to the results of the present study, it is possible to affirm that a more extended flap does not cause more postoperative symptoms and signs. On the contrary, achieving a better surgical view might potentially reduce the time necessary for the intervention and, therefore, reduce the severity of postoperative symptoms and signs. Further studies with larger sample sizes are needed to confirm such a consideration.

The present study presented some limits, including the small number of the subjects. Some limits were related to the methods used to assess postoperative signs. Indeed, while measuring trismus is a quite simple procedure, evaluating postoperative swelling is far more complicated due to the number of measures needed, and may be prone to errors. Further studies are needed to overcome these limits.

## Conclusion

Within the limits of the present study, it is possible to conclude that the duration of surgery and not the flap design might influence the acute postoperative symptoms and signs after lower third molar extraction.

## Acknowledgments

None.

## Authors’ contributions

NM attended to the study design and statistical analysis; RV and MV attended to the experimental part of the study; and GC and SC revised the entire study. NM drafted the manuscript. All authors critically revised the manuscript for intellectual content, and have read and approved the final version.

## Funding

No funds were requested for this study.

## Competing interests

The authors declare no competing interest with regards to authorship and/or publication of this paper.

## Ethics approval and consent to participate

The Ethics Committee of the District of Ferrara approved the study (IRB no. 3/2008), and each patient provided a written informed consent. The study was designed according to the Declaration of Helsinki.
